# Fenugreek seed proteins: Ultrasonic‐assisted extraction, characterization, and cupcake application

**DOI:** 10.1002/fsn3.4247

**Published:** 2024-06-17

**Authors:** Izzet Turker, Gamze Nur Olgun, Hilal Isleroglu

**Affiliations:** ^1^ Faculty of Engineering and Architecture, Food Engineering Department Tokat Gaziosmanpasa University Tokat Turkey

**Keywords:** coagulated protein, cupcake, fenugreek seeds, ultrasound‐assisted extraction, β‐Sheet

## Abstract

In this study, fenugreek seed proteins were extracted using ultrasonic‐assisted extraction with varying solid:solvent ratios (20–60 g/L) and sonication amplitudes (30%–80%) to determine optimal conditions for the highest extraction yield. The functional, structural, and nutritional characteristics of the protein isolates of fenugreek seeds were investigated. The highest yield (98.74 ± 0.49%) was achieved at a solid:solvent ratio of 43.83 g/L and an amplitude of 67.51%. The coagulated protein values of fenugreek seed protein isolates ranged from ~15.8% to 31.2%, water‐holding capacities ranged from ~2.2 to 3.2 g/g, oil‐holding capacities ranged from ~2.6 to 4.1 g/g, foaming capacities ranged from ~16.3% to 21.3%, foam stabilities ranged from ~59.7% to 78.1%, emulsion stabilities ranged from ~30.2 to 34.5 min, emulsion activities ranged from ~73.8 to 76.8 m^2^/g, and emulsion capacities ranged from ~26.9% to 30.5% under different extraction conditions. SDS‐PAGE analysis revealed three distinct bands (46, 59, and 80 kDa) for the protein isolates. FT‐IR spectroscopy showed a high presence of β‐sheet structures. The amino acid composition analysis of fenugreek seed protein isolates was determined, revealing richness in essential amino acids (317.97 g amino acid/kg protein isolate). In addition, cupcakes enriched with protein isolates (5%, 10%, and 20% as flour substitutes) were produced, and quality properties such as color change, browning index, moisture content, water activity, baking yield, bulk density, hardness, volume, symmetry, and uniformity indexes were determined. The application of protein isolates in cupcake production demonstrated the potential of fenugreek seeds as valuable ingredients for enhancing the nutritional profile of bakery products.

## Graphical abstract

Proteins extracted from fenugreek seeds using ultrasonic‐assisted extraction at different solid:solvent ratios and sonication amplitudes, and the optimum conditions ensuring the highest extraction efficiency, were determined. The functional, structural, and nutritional characteristics of the protein isolates of fenugreek seeds were investigated. Cupcakes were produced using protein isolates, and some quality properties of the cupcakes (color change, browning index, moisture content, water activity, baking yield, bulk density, hardness, volume, symmetry, and uniformity indexes) were determined.

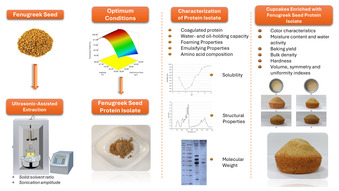

## 1 INTRODUCTION

The expanding market for plant proteins within the food industry presents a significant opportunity to address the nutritional requirements of the increasing global population while concurrently facilitating the shift toward more sustainable food systems. Consequently, there is considerable demand from consumers, industry stakeholders, and governmental bodies for nutritious food options enriched with plant‐based proteins (Hoehnel et al., 2019). Plant proteins, including fenugreek seed proteins, have emerged as a viable solution to address the escalating need for food proteins sustainably. Fenugreek seed proteins, with their nutritional qualities and functional properties, present a promising alternative to traditional protein sources.

Fenugreek (*Trigonella foenum graecum* L.) is a globally cultivated leguminous plant primarily valued for its spice. It belongs to the Leguminosae family and is characterized by small seeds. Various fenugreek races and species are distributed across Asia, Europe, Africa, and Australia, with historical cultivation spanning from Western Europe to China. Renowned for their medicinal properties, fenugreek seeds are commercially prized for their aromatic bitterness and are utilized in both culinary and therapeutic applications. Rich in protein, oils, and minerals, fenugreek seeds offer significant nutritional value, with lysine content comparable to soybean protein, thus meeting human protein requirements (Acharya et al., 2006; Srinivasan, 2006).

Traditional methods recover only about half of the proteins from substrates (Sun et al., 2023). Various studies have explored techniques to enhance protein extraction, including enzymatic and physical pretreatments. Ultrasound, among other physical methods, has gained attention for its ability to significantly boost extraction yields (Karki et al., 2010). The adoption of new extraction technologies enhances efficiency and improves techno‐functional and nutritional attributes, offering cost‐effective, safe, and eco‐friendly alternatives for plant protein production (Tiwari, 2015). However, industrial‐scale application remains limited despite these benefits (Liu et al., 2016). The choice of extraction method significantly impacts the functional properties of plant proteins, emphasizing the importance of selecting methods that preserve functionality (Pojić et al., 2018). Green extraction techniques, akin to those employed for phenolic compounds or volatile oils, are increasingly utilized for protein extraction. Ultrasonic‐assisted extraction (UAE) stands out as a pivotal method in the food industry.

UAE significantly enhances protein yield, influenced by factors like ultrasonic power, solid:solvent ratio, and extraction time (Pojić et al., 2018). UAE outperforms conventional methods, yielding higher protein levels from sources like chickpeas, defatted rice bran, and watermelon seeds (Gadalkar & Rathod, 2020; Ly et al., 2018; Wang et al., 2020). Optimization techniques, such as response surface methodology, further improve protein yield, particularly for rapeseed extraction (Yagoub, & Ma, & Zhou, 2017). UAE also enhances the solubility, emulsifying properties, foaming, and water‐holding capacity of protein isolates, surpassing conventional methods (Hu et al., 2013; Morales et al., 2015).

This study highlights the innovative use of ultrasonic‐assisted extraction (UAE) for obtaining protein isolates from fenugreek seeds, presenting a sustainable approach to food ingredient production. Proteins of fenugreek seeds were extracted using UAE under different process conditions, and the optimum conditions providing the highest extraction efficiency were determined. The proteins extracted under various conditions were thoroughly characterized, including their functional properties, molecular composition, and structural properties. The coagulated protein percentage, water‐holding capacity, oil‐holding capacity, foaming properties (foam capacity and foam stability), and emulsifying properties (emulsion activity, emulsion stability, and emulsion capacity) of protein isolates obtained under all extraction conditions in the UAE process were determined. The solubility properties, molecular weights, amino acid composition, and structural properties of protein isolates obtained under optimum extraction conditions were also determined. Moreover, cupcakes enriched with fenugreek seed protein isolates were produced, and some quality characteristics of the cupcakes were investigated.

## 2 MATERIALS AND METHODS

### 2.1 Material

After the elimination of extraneous materials from fenugreek seeds procured from a local market, the seeds underwent pulverization using a household grinder. The resulting powdered seed samples were sieved through a 630 μm mesh, followed by defatting of the under‐sieve samples using hexane. Subsequently, the defatted samples were air‐dried at 50°C for 12 h to eliminate residual hexane, yielding defatted fenugreek seed samples suitable for protein extraction. The total protein content of the defatted fenugreek seeds was determined to be 17.13 ± 0.10%.

### 2.2 Ultrasonic‐assisted extraction process and isolation of fenugreek seed proteins

In the UAE process for protein extraction from fenugreek seeds, a pH value of 11.47, determined from preliminary extractions at which protein solubility was the highest, was used and maintained. A ‘Central Composite Design’ was formulated to investigate the effects of solid:solvent ratio (20–60 g/L) and sonication amplitude (30%–80%) (Table 1). Sonication amplitude was expressed as a percentage, representing the longitudinal wave distance generated by the tip of sonicator. A laboratory‐scale sonicator with a ½“ diameter probe (Q 500, Q Sonica, 500 W, 20 kHz, USA) was used, where increasing amplitude led to higher cavitation intensity in the liquid. An α value of 0.8 was set to avoid excessive sample heating and calculated as follows: $\alpha =t_{\mathrm{on}}/\left(t_{\mathrm{on}}+t_{\mathrm{off}}\right)$, where *t*_on_ is the active sonication time (s) and *t* is the passive sonication time. To maintain the extraction temperature (25°C), a cooling bath setup was used. The samples were sonicated at various amplitude values (%) for 30 min and then centrifuged at 3250 *g* for 15 min to obtain the extract. The extraction yield was determined by dividing the protein content in the supernatant phase (extract) by the protein content of the initial powdered seed sample (Equation 1). Protein content was determined using the Kjeldahl method (AOAC, 2000) for isolates and powder samples and the Bradford method for supernatant phases (Feyzi et al., 2017).

(1) $$\text{Extraction yield}\;\left(\%\right)=\frac{\text{Protein amount of the extract}\;\left(g\right)}{\;\text{Protein amount of the fenugreek seeds}\;\left(g\right)}\times 100$$

**TABLE 1:** Experimental design and fenugreek seed protein extraction yields.

Solid‐to‐solvent ratio (g/L)	Amplitude (%)	Extraction yield (%)
60	30	51.10 (±1.07)
40	55	94.20 (±0.77)
40	55	94.72 (±0.59)
60	55	92.13 (±1.03)
20	30	61.24 (±0.59)
40	80	91.89 (±1.39)
40	55	96.01 (±0.59)
40	55	93.95 (±1.18)
40	55	94.72 (±1.18)
20	80	85.58 (±0.80)
60	80	98.48 (±0.79)
40	30	56.61 (±0.80)
20	55	92.53 (±1.56)

In the optimization process, the extraction yield served as the response variable, and the conditions yielding the highest extraction yield were determined using a desirability function approach. The regression model used for the analysis is presented in Equation 2.

(2) $$\text{Extraction yield}\;\left(\%\right)=\beta_{0}+\sum_{i=1}^{k}\beta_{i}X_{i}+\sum_{i=1}^{k}\beta_{\mathrm{ii}}X_{i}^{2}+\sum_{i=1}^{k-1}\sum_{j=i+1}^{k}\beta_{\mathrm{ij}}X_{i}X_{j}\;\left(k=1,2\right)$$

where, β_0_, β_*i*_, β_*ii*_, and β_*ij*_ are the coefficients, X is the independent variable, and *k* is the number of independent variables.

Following the extraction process, the pH values of the extracts were adjusted to 4.0 and then incubated at room temperature for 6 h. Subsequently, the samples underwent centrifugation at 7690 *g* for 60 min, after which the supernatant was decanted, and the precipitate was washed three times (5 min at 3250 *g*) using distilled water. The washed precipitates were then collected and lyophilized for 72 h using a Christ Alpha 1–4 LSC Plus lyophilizer (Germany). The powdered protein isolates obtained from lyophilization were stored in sealed tubes at −18°C until further analyses. The average recovery in the precipitation process was determined to be 93.75 ± 0.55% by quantifying the remaining protein in the supernatant phase after precipitation.

### 2.3 Characterization of the protein isolates

#### 2.3.1 Coagulated protein

Protein isolates' coagulated protein percentage was determined according to Kramer and Kwee (1977). Initially, 0.2 g of protein isolate was dissolved in 10 mL of a 0.025 M citrate–phosphate solution at pH 7.0, followed by centrifugation. The supernatant phase was treated with Biuret reagent and heated at 100°C for 15 min, twice. Absorbances before (A_1_) and after heating (A_2_) were measured at 540 nm, and % coagulated protein was calculated using Equation 3.

(3) $$\text{Coagulated protein}\;\left(\%\right)=\frac{A_{1}-A_{2}}{A_{1}}\times 100$$

#### 2.3.2 Water‐ and oil‐holding capacity

The water‐holding capacity and oil‐holding capacity were assessed with modifications to the method described by Vinayashree and Vasu (2021). Initially, 250 mg of protein isolate was vortexed with 15 mL of distilled water, left at room temperature for 1 h, and then centrifuged at 850 *g* for 20 min, with subsequent removal of the supernatant. The remaining sample was weighed to determine the water‐holding capacity (g water/g sample). For oil‐holding capacity, olive oil replaced water, with the resulting capacity expressed as grams of oil per gram of sample.

#### 2.3.3 Foaming properties

The foaming capacity and foam stability of the protein isolates were assessed (Timilsena et al., 2016). Aqueous solutions of the protein isolate (20 g/L) were homogenized using an Ultra‐Turrax IKA T‐18 Basic homogenizer (Germany) at 10,000 rpm for 5 min. Total volumes before (*V*_0_) and after (*V*_1_) homogenization were recorded. Foaming capacity, expressed as a percentage, was calculated by dividing the difference between *V*_1_ and *V*_0_ by *V*_0_. Foam stability was determined by measuring the total volume (*V*_2_) of the homogenized sample after 1 h at room temperature. Stability was calculated by dividing the difference between *V*_2_ and *V*_0_ by the difference between *V*_1_ and *V*_0_.

#### 2.3.4 Emulsifying properties

The emulsion activity and stability of the protein isolates were assessed using the modified turbidity method outlined by Feyzi et al. (2017). Initially, 22.5 mg of the sample was added to a 15 mL tube containing 4.5 mL of phosphate buffer solution (pH 7.0) and vortexed for 1 min. Then, 1.5 mL of sunflower oil was added and homogenized at 22,000 rpm for 2 min. For emulsion stability, 250 μL of the emulsion immediately post‐homogenization (*t* = 0) was mixed with 50 mL of 1 g/L sodium dodecyl sulfate solution. Absorbance at 500 nm was recorded as *A*_0_. Similarly, the absorbance of the emulsion kept at room temperature for 15 min (*t* = 15) was recorded as *A*_15_. Emulsion stability (min) was calculated using Equation 4.

(4) $$\text{Emulsion stability}\;\left(\min\right)=\frac{A_{0}}{\left(A_{0}–A_{15}\right)}\times t$$

For emulsion activity calculation, 800 μL of emulsion collected at *t* = 0 was dried in an oven at 120°C for 2 h to determine the oil volumetric fraction (Φ). Once a constant weight was achieved, the sample was weighed, and Φ was calculated using the oil's specific gravity (0.918 g/mL). Emulsion activity (m^2^/g) was determined using Equation 5.

(5) $$\text{Emulsion activity}\;\left(m^{2}/g\right)=\frac{2T\times D}{\Phi \times C}=\frac{\left(2\times 2.303\times A_{0}\times D\right)}{\left(\Phi \times C\times L\right)}$$

Here, *T* is turbidity (*T* = 2.303 × *A*_0_/*L*), *D* is dilution factor (200), Φ is volumetric fraction of oil (g oil/g sample), *C* is protein concentration in the solution (0.005 g/mL), and *L* is the path length of the cuvette (10^−2^ m).

The emulsion capacity was determined by adapting the method outlined by Neto et al. (2001). Initially, equal volumes of sunflower oil and protein isolate solutions (1.0% w/v) were combined and homogenized using an ultra‐turrax (7200 rpm, 2 min). These emulsions were then centrifuged at 1000 *g* for 2 min. The height of the emulsified layer (*H*_1_) and the total height of the emulsion before centrifugation (*H*_0_) were measured. Emulsion capacity was calculated as the ratio of *H*_1_ to *H*_0_, expressed as a percentage.

#### 2.3.5 Protein solubility

The solubility of the protein isolates (g/L) was determined using the method described by Feyzi et al. (2015). The pH values of the protein isolate solutions, prepared with distilled water at a concentration of 15 g/L, were adjusted to values ranging from 2.0 to 12.0 using HCl or NaOH. After agitating the samples for 30 minutes at room temperature, they were centrifuged at 3250 *g* for 15 min to determine the protein content in the supernatant phase.

#### 2.3.6 Determination of molecular weight

The determination of protein molecular weights in the fenugreek seed protein isolate obtained under optimum conditions was carried out using the SDS‐PAGE (Sodium Dodecyl Sulfate‐Polyacrylamide Gel Electrophoresis) method (Laemmli, 1970). SDS‐PAGE analysis was conducted using vertical electrophoresis and the Coomassie staining technique. 12% gels were utilized, and each sample was loaded with 10 and 50 μg. Electrophoresis was performed at 200 V for 50 min, followed by staining the gel with Coomassie Brilliant Blue R‐250. A ‘Thermo Fischer PageRuler Prestained Protein Ladder’ containing molecular weights ranging from ~10 to 180 kDa served as a standard. The gel images were processed using Microsoft 365 PowerPoint (USA), where specific lanes containing Sample (50 μg) and Sample (10 μg) were cut, spliced, and re‐colored for figure construction.

#### 2.3.7 Determination of the structural properties

The structural properties of the fenugreek seed protein isolate obtained under optimum conditions were examined utilizing Fourier transform infrared (FT‐IR) spectrometry (Perkin Elmer 400, USA). The analysis employed the Diamond Attenuated Total Reflectance (ATR) method, with measurements conducted across the spectrum range of 4000–400 cm^−1^. Protein secondary structures were assessed by focusing on the Amid I band region (1600–1700 cm^−1^) in FT‐IR spectra, and analysis was performed using the Peakfit v4.12 software package (Systat Software, USA).

#### 2.3.8 Amino acid composition

To ascertain the amino acid composition, acid hydrolysis was employed on the fenugreek seed protein isolate obtained under optimal conditions. The amino acid profiles of the samples were analyzed using ultra‐fast liquid chromatography (UFLC) with ultraviolet detection (UV) (Shimadzu 20A, Japan). The analysis encompassed the determination of aspartic acid, glutamic acid, serine, glycine, histidine, arginine, threonine, alanine, proline, tyrosine, valine, methionine, isoleucine, leucine, phenylalanine, and lysine. For the quantification of tryptophan content, high‐performance liquid chromatography with fluorescence detection (HPLC‐FLD) (Shimadzu 20A, Japan) was employed. Amino acid scores were computed by comparing the amino acid values of the fenugreek seed protein isolate to those of the designated protein reference.

### 2.4 Production of cupcakes using fenugreek seed protein isolate

Cupcake production was carried out using the protein isolate obtained under optimum conditions. The formulation of the cupcake without protein isolate produced as the control group was given as follows: 23.5% flour, 20% sugar, 18% egg, 18% sunflower oil, 1% baking powder, and 19.5% water. To produce cupcakes supplemented with protein isolate, the amount of flour in the given formulation was replaced with 5%, 10%, and 20% protein isolate. The prepared batter (~40 g) was transferred to aluminum cupcake molds and baked in a convection oven at 180°C for 15 min.

#### 2.4.1 Determination of the color characteristics of batter and cupcakes

The color properties of the batter and crumb and crust of the cupcakes were determined by measuring the CIE L*, a*, and b* values (Minolta, CR‐300). The total color difference (*ΔE*) in the crumb and crust of the cupcakes was calculated using Equation 6, and the browning index (BI) of the cupcakes was calculated using Equation 7.

(6) $$\mathrm{\Delta E}=\sqrt{{\left(\mathrm{\Delta L}*\right)}^{2}+{\left(\mathrm{\Delta a}*\right)}^{2}+{\left(\mathrm{\Delta b}*\right)}^{2}}$$

Here, ΔL* *= L*_s_*‐L_0_*, Δa* *= a*_s_*‐a_0_*, Δb* *= b*_s_*‐b_0_*, and subscript ‘0’ denotes the values determined for the batter of the control sample, and subscript ‘s’ denotes the values determined for the cupcakes enriched with fenugreek seed protein isolate.

(7) $$\mathrm{BI}=\frac{\left[100\times \left(\frac{a^{*}+1.79L^{*}}{5.645L^{*}+a^{*}-3.012b^{*}}-0.31\right)\right]}{0.17}$$

#### 2.4.2 Determination of moisture content and water activity of cupcakes

The moisture content of the cupcakes was determined at 70°C using an infrared moisture analyzer (Shimadzu MOC‐63 U, Japan). The water activity (*a*_*w*_) values of the cupcakes were measured using a water activity measurement device (AquaLab Model Series 3TE, USA). The average moisture content of the batter used for cupcake production was determined to be 35.5 ± 0.4%, while the average water activity was 0.937 ± 0.006.

#### 2.4.3 Determination of baking yield

The baking yield for cupcakes baked under the same conditions was calculated by dividing the weight of the baked cupcakes by the weight of the batter placed in the mold and expressed as a percentage (Majzoobi et al., 2018).

#### 2.4.4 Determination of bulk density

The bulk density (kg/m^3^) of cupcakes was calculated by dividing the weight of the cupcake by its volume. Cupcake weights were measured using a precision scale, while volume was determined using the displacement principle with rapeseed (AACC, 2000).

#### 2.4.5 Determination of hardness

Hardness analysis for cupcake samples was conducted using a hardness testing device (Zwick z0.5, Germany) (Wirkijowska et al., 2020). The produced cupcakes were placed under the cylindrical probe (37.9 mm diameter). The trigger force in the device was set to 0.03 N, and the compression distance (ΔL) of the sample was set to 25 mm. The speed was set to 35 mm/min. Once the compression process was completed, the results were read and recorded in Newtons (N). All analyses were conducted in triplicate.

#### 2.4.6 Determination of volume, symmetry, and uniformity indexes

Mechanical calipers measured the volume, symmetry, and uniformity indexes of the cupcakes. Each cupcake was vertically sliced from its center using a knife. Measurements included the cupcake's vertical height and heights at points 60% from the cake's central point to its right and left sides using a millimeter template. The volume index summed up these heights. The symmetry index compared twice the cupcake's vertical height to the sum of the heights at these points. The uniformity index was determined by subtracting the left‐side height from the right‐side height at the 60% points (AACC, 2000).

### 2.5 Statistical analysis

The one‐sample *t*‐test and “Univariate Variance Analysis, Duncan post hoc” test were conducted utilizing the SPSS 21.0 software package. Regression analysis and optimization processes aimed at determining the effects of all process variables were executed using the Design Expert 7.0 software (Stat‐Ease, Inc., USA).

## 3 RESULTS AND DISCUSSION

### 3.1 Ultrasonic‐assisted extraction of fenugreek seed proteins

In the UAE process applied for the extraction of proteins from fenugreek seeds, the solvent pH value (pH 11.47), determined according to the preliminary tests where the solubility of proteins was the highest, was kept constant. The experimental design used for the UAE process and the resulting extraction yields are presented in Table 1. It was observed that low extraction yield values were obtained at points where the ultrasonication amplitude was low (30%). According to the results, the highest extraction yield was achieved with a yield of 98.48 ± 0.79% under the conditions of a 60 g/L solid:solvent ratio and 80% amplitude. The lowest extraction yield was determined to be 51.10 ± 1.07% under the conditions of a 60 g/L solid:solvent ratio and 30% amplitude (Table 1). With the UAE process, the extraction yield reached approximately 98% within 30 min, which can be considered a short extraction time. The efficiency of the UAE process can be explained by the increase in the contact surface area between the solid and solvent due to mechanical vibrations occurring in the extraction medium. Additionally, effective extraction is achieved because ultrasonication disrupts the cell wall, breaks molecular bonds, and increases mass transfer rates (Görgüç et al., 2019). Chemat et al. (2017) emphasized that the UAE process shortens extraction time, reduces energy consumption, decreases solvent usage, increases solution homogeneity, reduces temperature gradient, and provides better process control in protein extraction. In their study on pea protein extraction, Wang et al. (2020) increased the extraction yield from approximately 72% in classical extraction to approximately 83% in 13.5 min with the UAE process, compared to 30 min in classical extraction. In a study on wheat seed protein extraction, Zhu et al. (2009) found that the extraction yield increased by 37%–57% with the use of an ultrasonic probe compared to the classical extraction process at the same extraction time. Similarly, Karki et al. (2010) reported a 46% increase in yield using an ultrasonic probe in soybean protein extraction compared to the classical extraction process. Li et al. (2017) found that the use of an ultrasonic probe resulted in a 90% total protein yield from rice powder. When studies in the literature are examined, it is generally seen that the UAE process increases protein extraction efficiency, shortens extraction time, and reduces solvent use compared to classical extraction.

For the optimization of protein extraction from fenugreek seeds using the UAE process, extraction yield was selected as the dependent variable, and a second‐degree polynomial model was created. According to the ANOVA table provided in Table 2, the generated polynomial model is statistically significant (*p* < .05), while the lack of fit of the model is statistically insignificant (*p* > .05). The linear and quadratic effects of the solid:solvent ratio were found to be statistically insignificant (*p* > .05). However, it was determined that the linear and quadratic effects of ultrasonication amplitude are statistically significant (*p* < .05). Furthermore, the interaction between the solid:solvent ratio and amplitude has a statistically significant effect on the extraction yield (*p* < .05) (Table 2). The model equation written in terms of the actual values of the factors is provided in Equation 8. The model's *R* ^2^, adjusted *R* ^2^, adequate precision, predicted residual sum of squares, and coefficient of variation values for extraction yield are also shown in Table 2. The suitability of the model was further confirmed with statistical data. Additionally, the proximity of *R* ^2^ and adjusted *R* ^2^ values indicates that the model does not contain statistically insignificant terms.

(8) $$\text{Extraction yield}\;\left(\%\right)=-19.32+3.66X_{2}+0.01X_{1}X_{2}-0.03X_{2}^{2}$$

**TABLE 2:** ANOVA table and statistical values.

Source	DF	Sum of squares	*F* value	*p*‐Value
Model	5	3323.70	650.23	<.0001
X_1_	1	0.93	0.91	.3715
X_2_	1	1907.73	1866.08	<.0001
X_1_X_2_	1	132.77	129.87	<.0001
X_1_^2^	1	4.71	4.61	.0690
X_2_^2^	1	1038.19	1015.53	<.0001
Residual	7	7.16		
Lack of fit	3	4.64	2.45	.2030
Pure error	4	2.52	650.23	<.0001
Total	12	3330.85		

*Note*: *R* ^2^: .9979, adj‐*R* ^2^: .9963, Adequate precision: 68.692, PRESS: 39.22, C.V. (%): 1.01. X_1_: Solid‐to‐solvent ratio (g/L), X_2_: Amplitude (%) (°C), DF: Degrees of freedom, Adj‐*R* ^2^: Adjusted *R* ^2^, PRESS: Predicted residual error sum of squares, C.V. (%): Coefficient of variation.

**FIGURE 1:**
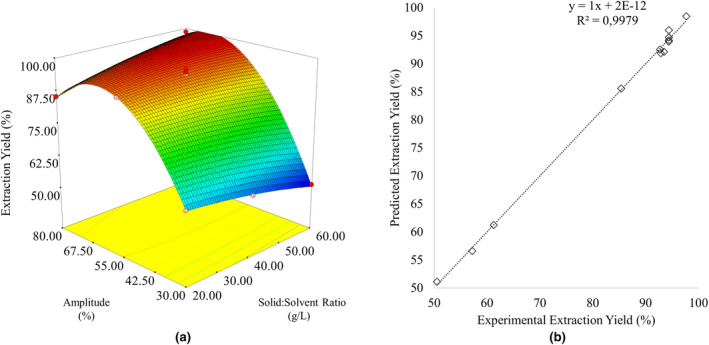
(a) Response surface graph and (b) the relation between predicted and experimental extraction yield values.

Optimization of protein extraction from fenugreek seeds using the UAE process was determined using the desirability function approach. It was found that the highest desirability value (*d* = 1.0) was achieved when the solid:solvent ratio was 43.83 g/L and the amplitude was 67.51%. These conditions were selected as the optimum extraction process conditions. There was no statistically significant difference (*p* > .05) between the predicted protein extraction yield (99.06%) and the experimental verification test results (98.74 ± 0.49%) under these conditions. The response surface graph showing the effects of independent variables (Figure 1a) and the graphical representation showing the relationship between the predicted and experimental data (Figure 1b) are presented in Figure 1. The adequacy of the generated model was confirmed by the closeness of the predicted and experimental values.

### 3.2 Characterization of fenugreek seed proteins

#### 3.2.1 Coagulated protein

The coagulated protein values of fenugreek seed proteins obtained by the UAE process varied between 15.82% and 31.17%. It was observed that as the amplitude value increased in the UAE process, the percentage of coagulated protein also increased (*p* < .05) (Table 3). It has been reported that ultrasonication increases the coagulation properties of proteins (Akdeniz & Akalın, 2019). The high percentages of coagulated protein in the proteins obtained by the UAE process indicate that these protein isolates can be used in gel and foam formation (Kramer & Kwee, 1977).

**TABLE 3:** Functional properties of the protein isolates.

Ampl. (%)	S:S (g/L)	Foaming capacity (%)	Foam stability (%)	Water‐holding capacity (g/g)	Oil‐holding capacity (g/g)	Coagulated protein (%)	Emulsion stability (min)	Emulsion activity (m^2^/g)	Emulsion capacity (%)
30	20	16.33 (±0.58)^d^	75.49 (±0.85)^a^	3.14 (±0.02)^a^	2.65 (±0.05)^fg^	16.64 (±0.17)^e^	30.20 (±0.15)^e^	74.86 (±0.17)^d^	27.11 (±0.44)^cd^
	40	18.33 (±0.58)^c^	74.56 (±2.88)^a^	3.07 (±0.02)^b^	2.60 (±0.02)^g^	16.06 (±0.33)^f^	30.40 (±0.19)^e^	73.83 (±0.22)^f^	26.89 (±0.22)^d^
	60	16.67 (±0.58)^d^	78.06 (±2.76)^a^	3.15 (±0.02)^a^	2.69 (±0.06)^f^	15.82 (±0.16)^f^	30.31 (±0.24)^e^	76.63 (±0.11)^a^	27.85 (±0.13)^c^
55	20	20.33 (±1.53)^ab^	68.87 (±0.99)^b^	2.96 (±0.03)^d^	3.01 (±0.02)^e^	21.60 (±0.41)^d^	31.88 (±0.15)^c^	74.14 (±0.14)^e^	29.33 (±0.44)^b^
	40	21.33 (±0.58)^ab^	74.96 (±3.11)^a^	2.96 (±0.01)^d^	3.16 (±0.01)^d^	24.28 (±0.34)^c^	31.83 (±0.17)^c^	75.03 (±0.07)^cd^	30.15 (±0.68)^a^
	60	19.00 (±1.00)^bc^	68.55 (±3.61)^b^	3.02 (±0.03)^c^	3.15 (±0.03)^d^	26.03 (±0.34)^b^	30.78 (±0.04)^d^	76.79 (±0.25)^a^	28.67 (±0.38)^b^
80	20	20.67 (±0.58)^a^	59.68 (±2.40)^c^	2.18 (±0.01)^g^	4.00 (±0.06)^b^	30.72 (±0.17)^a^	34.50 (±0.17)^a^	76.04 (±0.07)^b^	30.52 (±0.64)^a^
	40	21.00 (±1.00)^a^	62.00 (±2.96)^c^	2.32 (±0.03)^f^	3.97 (±0.02)^c^	30.87 (±0.16)^a^	33.14 (±0.21)^b^	74.94 (±0.10)^d^	30.22 (±0.22)^a^
	60	20.00 (±1.00)^ab^	66.70 (±1.71)^b^	2.38 (±0.04)^e^	4.09 (±0.02)^a^	31.17 (±0.25)^a^	34.41 (±0.19)^a^	75.27 (±0.14)^c^	30.52 (±0.68)^a^
Optimum Point 67.51%, 43.83 g/L		21.67 (±0.58)	69.26 (±1.87)	2.68 (±0.01)	3.85 (±0.02)	31.03 (±0.25)	34.65 (±0.28)	73.86 (±0.27)	31.19 (±0.26)

Abbreviations: Ampl., Amplitude (%); S:S, Solid‐to‐solvent ratio.

^a–g^Means with unusual superscripts within a column are significantly different (*p* < .05).

#### 3.2.2 Water‐ and oil‐holding capacity

While the water‐holding capacity values of fenugreek seed protein isolates obtained by the UAE process varied between 2.18 and 3.15 g/g, the value for the protein isolate produced under optimum conditions was determined to be 2.68 ± 0.01 g/g. It was observed that water‐holding capacity values increased significantly when low amplitude values were used in the UAE process (*p* < .05) (Table 3). It has been reported that when high power or high amplitude values are used in the UAE process, water‐holding capacity values may decrease due to particle size reduction (Mir et al., 2019). In other studies, the water‐holding capacity value of fenugreek seed protein concentrate was 1.56 g/g (El Nasri & El Tinay, 2007), while that of fenugreek seed protein isolate was 2.70 g/g (Feyzi et al., 2015).

The oil‐holding capacity values of fenugreek seed protein isolates obtained by the UAE process ranged from 2.60 to 4.09 g/g. It was determined that the highest oil‐holding capacity values were obtained under conditions where the amplitude value was 80% (*p* < .05) (Table 3). The oil‐holding capacity of the protein isolate obtained under optimum conditions during the UAE process was determined to be 3.85 ± 0.02 g/g. The increase in oil‐holding capacity due to the UAE process can be attributed to the exposure of hydrophobic groups in the protein structure to ultrasonication (Resendiz‐Vazquez et al., 2017).

#### 3.2.3 Foaming properties

The foam capacities of fenugreek seed protein isolates obtained by the UAE process were determined to be in the range of 16.33%–21.33%, and foam stabilities ranged from 59.68% to 78.06%. An increase in foam stability values was found to be statistically significant when a 30% amplitude value was used (*p* < .05). It was observed that the foam stability, which showed a statistically significant increase at low sonication amplitude values, decreased as the amplitude increased (*p* < .05) (Table 3). The foam capacity of the protein isolate obtained under optimum conditions during the UAE process was determined as 21.67% ± 0.58, and the foam stability was determined as 69.26% ± 1.87. Feyzi et al. (2017) reported a 19% foam capacity value and a 65% foam stability value for fenugreek seed protein isolate. The higher foam capacity of the protein isolates obtained at the optimum point with the UAE process can be attributed to ultrasonication, which exposes hydrophobic groups in the proteins and causes partial denaturation of the protein structure (Wang et al., 2020).

#### 3.2.4 Emulsifying properties

The emulsion stability of fenugreek seed protein isolates obtained by the UAE process was found to vary between 30.20 and 34.50 min (Table 3). The emulsion stability value for protein isolates obtained under optimum conditions during the UAE process was found to be 34.65 ± 0.28 min. Feyzi et al. (2017) determined the emulsion stability values for protein isolates extracted from fenugreek seeds using different solvents in the range of 11.59–22.23 min.

Feyzi et al. (2017) determined the emulsion activity values for protein isolates extracted from fenugreek seeds in the range of 70.00–93.28 m^2^/g. In our study, the ultrasonication process is thought to increase molecular flexibility, leading to more effective adsorption of protein molecules at the water–oil interface. The emulsion activities of fenugreek seed protein isolates obtained by the UAE process were determined to be in the range of 73.83–76.79 m^2^/g (Table 3). The emulsion activity of protein isolates obtained under optimum conditions during the UAE process was determined to be 73.86 ± 0.27 m^2^/g. The emulsion activity values of fenugreek seed protein isolates obtained by the UAE process were found to be higher than those of soy protein (L'Hocine et al., 2006) and pea protein (Wang et al., 2020), but lower than flaxseed protein (Kaushik et al., 2016).

The emulsion capacities of fenugreek seed protein isolates obtained by the UAE process were found to vary between 26.89% and 30.52% (Table 3). The emulsion capacity of protein isolates obtained under optimum conditions during the UAE process was determined to be 31.19% ± 0.26. Feyzi et al. (2017) reported the highest emulsion capacity for fenugreek seed protein isolate was at pH 3.0 (39%) and pH 10 (34%). The obtained results are in line with the literature.

#### 3.2.5 Protein solubility

**FIGURE 2:**
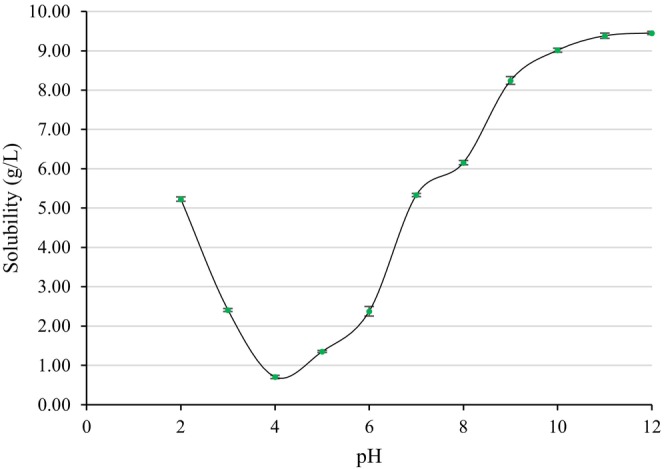
Solubility of fenugreek seed protein isolate.

Protein solubility is related to molecular size and structure. Hu et al. (2013) reported that ultrasonication reduces particle size, leading to increased interaction between protein and water molecules. The solubility of fenugreek seed protein isolates extracted under the optimum conditions of the UAE process is presented in Figure 2. According to the results, the solubility values of the protein isolate obtained under the optimum conditions of the UAE process (43.83 g/L solid:solvent ratio, 67.51% amplitude) varied between 0.70 and 9.45 g/L in the pH range of 2.0 to 12.0. The lowest solubility values were observed at pH 4.0. Similarly, Feyzi et al. (2017) reported an isoelectric point for fenugreek seed proteins at pH 4.5. At the isoelectric point, a balance is established between negatively and positively charged ions, resulting in a net charge of zero. Consequently, due to the decrease in electrostatic repulsion forces, proteins lose their solubility and precipitate because of hydrophobic interactions. On the other hand, the electrostatic repulsion forces between charged ions outside the isoelectric point promote the dissolution of proteins under acidic and alkaline conditions, which can vary for each protein (Singh et al., 2005). The highest solubility values were obtained at pH 11.0 and pH 12.0 (Figure 2). The improved solubility of fenugreek seed proteins at high pH values can be explained by the increased repulsive force of negatively charged ions, which prevents the formation of protein aggregates (Feyzi et al., 2015). It is speculated that one of the reasons for the high solubility of protein isolates obtained by the UAE process in this study could be particle size. In studies involving soy protein and black bean protein, the application of ultrasonication has been shown to enhance the solubility of proteins (Hu et al., 2013; Jiang et al., 2014).

#### 3.2.6 Structural properties

**FIGURE 3:**
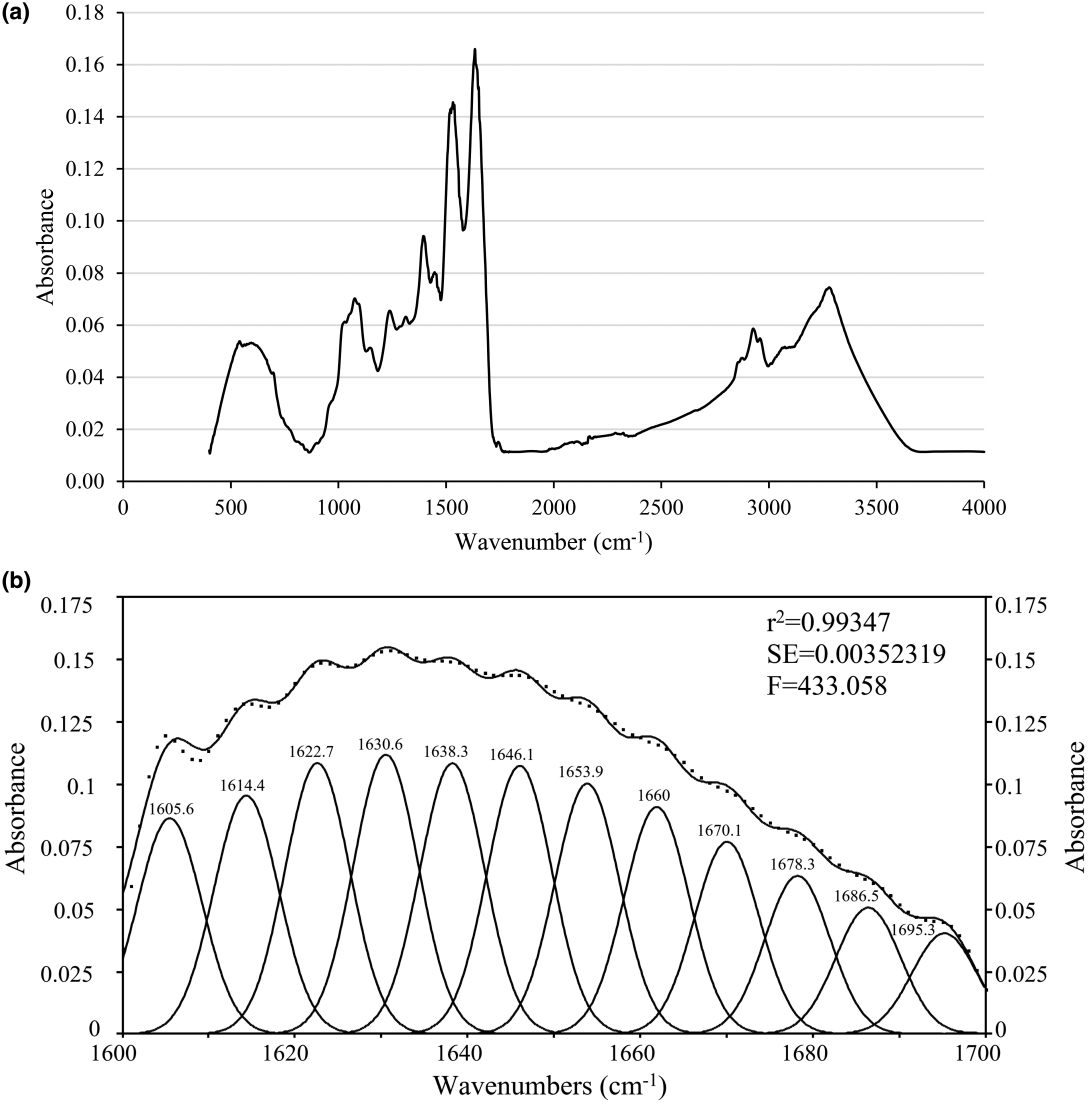
(a) FT‐IR spectra of fenugreek seed protein; (b) determination of the peak areas in the Amide I band.

The structural characteristics of fenugreek seed protein isolates obtained via UAE and precipitation at the isoelectric point were analyzed using Fourier transform infrared spectroscopy (FT‐IR). FT‐IR detects molecular vibrations, generates spectral peaks that are indicative of specific functional groups (Barth, 2007). The FT‐IR spectrum of the fenugreek seed protein isolate exhibited characteristic bands of protein structures, including the Amid I (1600–1700 cm^−1^), Amid II (1480–1585 cm^−1^), and Amid III (1200–1400 cm^−1^) regions, indicating peptide bond stretching, N–H bending, and interactions with carbohydrates (Barth & Zscherp, 2002; Carbonaro et al., 2008; de la Rosa‐Millán et al., 2018; Kong & Yu, 2007) (Figure 3a). Additionally, peaks in the Amid A (~3200–3500 cm^−1^) and Amid B (~2925 cm^−1^) regions indicate protein–water interactions and C–H stretching vibrations of lipids, proteins, and carbohydrates (Barth, 2007; Feyzi et al., 2017).

The determination of secondary structures was based on the Amid I band region, where α‐helix, β‐sheet, random coil, or β‐turn components are identified (Barth, 2007) (Figure 3b). Thirteen peaks in this region were analyzed, and the β‐sheet structure predominantly revealed potential thermal stability (Wang et al., 2020). The percentages of the α‐helix, β‐sheet, random coil, β‐turn, and side chains were determined as 20.00, 42.62, 10.90, 20.57, and 5.92, respectively. Similar studies on protein isolates obtained from soy seeds also found that β‐sheet structures are dominant (Wang et al., 2011). These findings contribute to our understanding of the structural properties of fenugreek seed protein isolates and their potential applications in the food and industrial sectors.

#### 3.2.7 Amino acid composition

The results from the amino acid composition analysis are presented in Table 4. The total essential amino acid content of the fenugreek seed protein isolate was determined to be 317.97 g amino acid/kg protein isolate, with a high level of lysine and a low level of histidine and methionine (Table 4). In general, considering the low lysine, high histidine, and methionine content of cereal products (Galili & Amir, 2013), it is thought that the use of fenugreek seed protein isolates in bakery products such as cakes, bread, and snacks could improve the nutritional quality. It was observed that leucine and lysine, which are essential amino acids, and arginine and glutamic acid, which are non‐essential amino acids, were higher than other amino acids. Similar results have been reported by Feyzi et al. (2015) for fenugreek seed protein isolate, highlighting its richness in lysine and its low content of histidine and methionine. Gorissen et al. (2018) determined the amino acid compositions of protein isolates commercially produced from different plant sources (soy, corn, flaxseed, and rice) and reported that the methionine content in plant‐based proteins was lower than in animal‐based proteins. Although previous studies have reported low levels of essential amino acids and leucine in protein isolates obtained from different sources, this study found that the fenugreek seed protein isolate had high levels of leucine and essential amino acids (Table 4). This finding not only demonstrates the nutritional quality of fenugreek seed protein isolate, but also indicates its potential for use in new and functional products.

**TABLE 4:** Amino acid composition of fenugreek seed protein isolate.

Amino acid	FEPI	Hen's egg	Soy protein isolate	Amino acid score
Essential amino acids (g amino acid/100 g protein isolate)				
Histidine	1.68	2.40	2.53	0.70
Threonine	2.71	5.10	3.86	0.53
Valine	4.77	7.50	4.80	0.64
Methionine	1.82	3.20	1.26	0.57
Phenylalanine	4.59	5.10	4.94	0.90
Isoleucine	4.09	5.60	4.54	0.73
Leucine	6.27	8.30	7.78	0.76
Lysine	5.87	6.20	6.38	0.95
*Total*	31.79	46.40	36.09	—
Non‐essential amino acids (g amino acid/100 g protein isolate)				
Tyrosine	2.76	4.00	3.14	0.69
Aspartic acid	1.79	10.70	11.70	0.17
Glutamic acid	4.67	12.00	18.70	0.39
Serine	2.39	7.90	5.49	0.30
Glycine	3.29	3.00	4.18	1.10
Arginine	4.72	6.10	7.23	0.77
Alanine	4.05	5.40	4.26	0.75
Proline	3.89	3.80	5.49	1.02
Cysteine	—	1.80	1.33	—
Tryptophan	1.10	—	1.28	—
Total	28.65	54.70	62.80	—

Abbreviation: FEPI, Fenugreek seed protein isolate.

Amino acid scores were calculated using the amino acid values of the reference protein (Hen's Egg) in Table 4. The amino acid scores ranged from 0.17 to 1.10. The amino acid scores of essential amino acids were close to those of hen's egg, except for methionine and threonine. Moreover, the amino acid values of fenugreek seed protein were compared with those of soy protein isolate reported by FAO (1992). The essential amino acid values of the fenugreek seed protein isolate were found to be close to those of the soy protein isolate, except for threonine.

According to FAO's provisional amino acid scoring model, amino acid scores are given in g amino acid/100 g sample. The values for leucine, isoleucine, lysine, methionine + cysteine (sulfur‐containing amino acids), phenylalanine + tyrosine, threonine, tryptophane, and valine were given as 6.27, 4.09, 5.87, 1.82, 7.35, 2.71, 1.10, and 4.77 g amino acid/100 g sample, respectively. The amino acid scores of the fenugreek seed protein isolate for leucine, isoleucine, lysine, methionine + cysteine (sulfur‐containing amino acids), phenylalanine + tyrosine, threonine, and tryptophane were calculated as 0.90, 1.02, 1.07, 0.52, 1.22, 0.68, 1.10, and 0.95, respectively. Except for sulfur‐containing amino acids (methionine + cysteine, 0.52), good scores were obtained. The lysine score of fenugreek seed protein isolate was 1.07, indicating that fenugreek seed protein isolate can be combined with cereal‐based products typically low in lysine. Fenugreek seed protein isolate may have potential for the development of new and functional products and can be used in food supplements.

#### 3.2.8 Molecular weight

**FIGURE 4:**
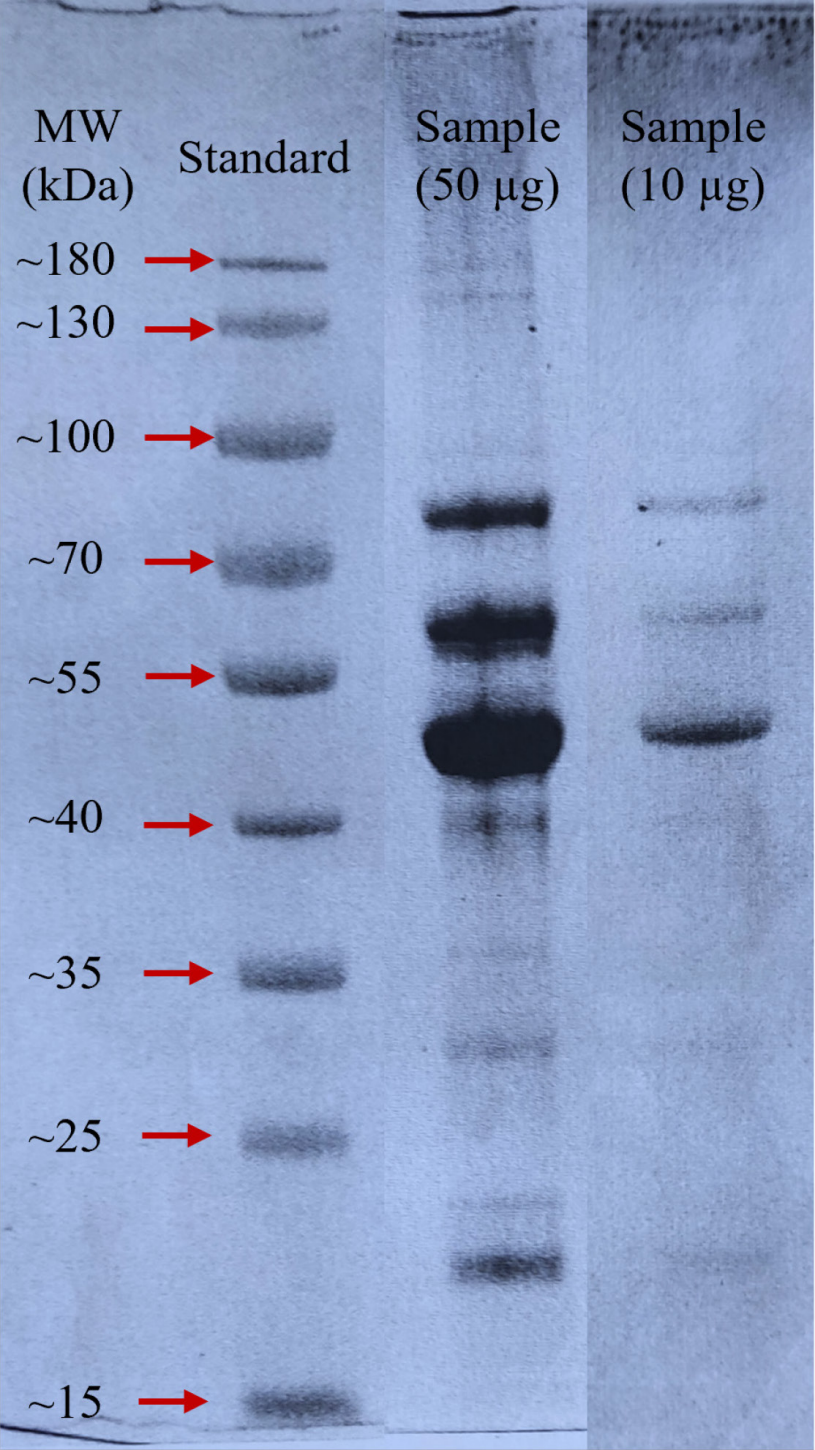
SDS‐PAGE gel image.

The SDS‐PAGE gel is shown in Figure 4. The protein isolate obtained from fenugreek seeds exhibited approximately 10 bands at various molecular weights, including 175, 159, 80, 59, 46, 38, 31, 27, 23, and 22 kDa. Notably, three distinct bands were exhibited in the protein isolate; the most prominent ones were identified at approximately 80, 59, and 46 kDa. Bands between 22 and 70 kDa are associated with globulins, particularly legumins and vicilins, which are the primary protein constituents in legumes (Feyzi et al., 2017). The bands at 46 kDa could be attributed to α‐legumin in the fenugreek seed protein isolate. Additionally, bands ranging from 50 to 80 kDa are attributed to vicilin and covicilin, with predominant bands observed at ~59 and 80 kDa, representing polypeptide constituents of vicilin and convicilin (Berrazaga et al., 2020).

### 3.3 Cupcake properties enriched with fenugreek seed protein isolate

#### 3.3.1 Color characteristics of batter and cupcakes

**FIGURE 5:**
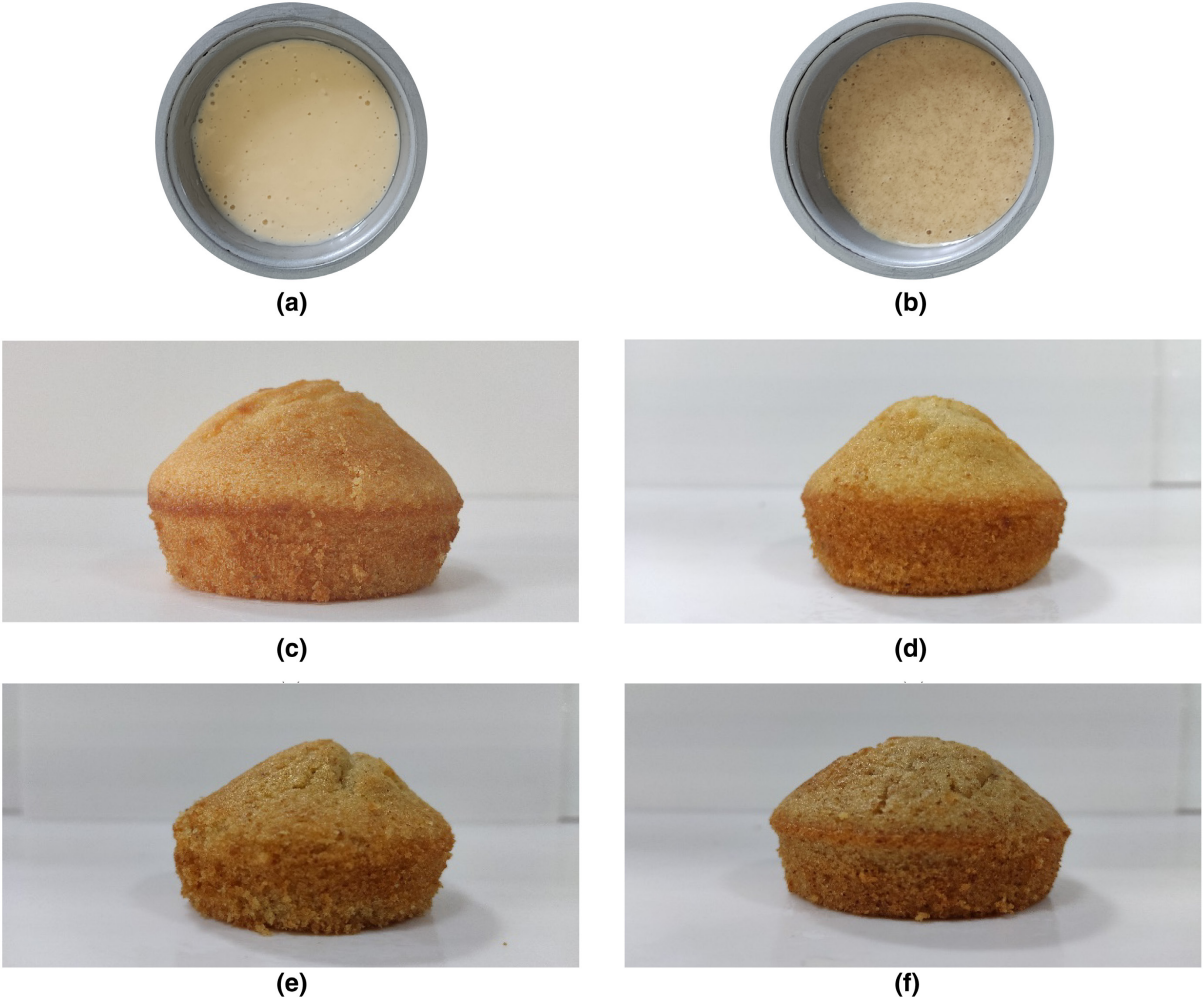
Images of batters and cupcakes: (a) control batter, (b) 10% protein isolate added batter, (c) control, (d) 5%, (e) 10%, and (f) 20% fenugreek seed protein isolate addition as a flour substitute.

The color properties of the batter and cupcake crumb and crust were analyzed. It was observed that as the proportion of protein isolate increased, there was a decrease in L* values for the batter, while a* values increased. Similarly, b* values increased with the addition of protein isolate. The same trend was observed for cupcake crumb and crust samples, with the highest L* value in the control sample and the lowest in the sample with 20% protein isolate. Visual representations of the differences are shown in Figure 5.

Table 5 presents the total color change (ΔE) values calculated for the cupcake crumb and crust. When the ΔE values obtained for the cupcake crusts were examined, it was observed that the values increased with the increase in protein content (*p* < .05). The highest ΔE values for both the crumb and crust were obtained in cupcakes added with 20% UAE‐treated protein isolate, while the lowest values were determined in control samples (*p* < .05) (Table 5). The browning index (BI) is a parameter that indicates the degree of browning in the crust and reflects the occurrence of enzymatic and/or non‐enzymatic browning reactions (Palou et al., 1999). Generally, the crust color of cakes is shaped by Maillard and caramelization reactions that occur during baking. In this study, it was found that the BI values of protein isolate‐added cupcakes were higher than for the control samples (*p* < .05) (Table 5). It is thought that dark‐colored compounds formed as a result of reactions occurring between reducing sugars and free amino groups at high temperatures contribute to the darkening of the crust color of the cupcakes. Similar results have been obtained for cakes formulated with soy protein isolate (Majzoobi et al., 2014).

**TABLE 5:** Color change, browning, volume, symmetry, and uniformity index values.

Sample	*ΔE*		BI	Volume index (mm)	Symmetry index (mm)	Uniformity index (mm)
	Crumb	Crust				
Control	15.27 (±0.68)^d^	29.78 (±0.92)^d^	116.07 (±3.78)^c^	106.20 (±0.32)^a^	24.23 (±0.47)^a^	1.28 (±0.33)^a^
PI 5%	17.42 (±1.96)^c^	34.05 (±1.23)^c^	137.92 (±5.93)^a^	103.83 (±0.17)^b^	24.35 (±0.47)^a^	0.55 (±0.06)^bc^
PI 10%	20.54 (±0.61)^b^	34.93 (±0.66)^ab^	138.84 (±3.33)^a^	100.93 (±0.15)^c^	23.05 (±0.30)^b^	0.35 (±0.19)^cd^
IP 20%	27.54 (±1.50)^a^	35.51 (±0.77)^a^	128.94 (±4.76)^b^	99.60 (±0.32)^d^	22.05 (±0.10)^c^	0.10 (±0.00)^d^

*Note*: ^a–d^Means with unusual superscripts within a column are significantly different (*p* < .05).

Abbreviations: BI, Browning index; PI, Protein isolate.

#### 3.3.2 Volume, symmetry, and uniformity indexes

The volume, symmetry, and uniformity index values of cupcakes with added protein isolate are presented in Table 5. The volume index is an indicator of the amount of air trapped in the interior of the cake after baking (Zhou et al., 2011). According to the results obtained, it was determined that the volume index values of cupcakes to which protein isolate was added were lower than the control group samples (*p* < .05). Additionally, it is observed that as the protein isolate ratio increases, the volume index values decrease (Table 5). The swelling of the cake occurs because of the entrapment of air bubbles formed during the whipping of eggs and sugar within the structure formed by the flour during baking (Majzoobi et al., 2014). It is thought that the decrease in volume index values due to the increasing protein isolate ratios in the formulation may be attributed to the decrease in the amount of gluten in the structure with the decrease in flour and the weakening of the structure that traps air bubbles. Similar results have been observed in studies where pomegranate peel powder is added instead of flour in cake production and pomegranate husk is added in bread production (Bhol et al., 2016). The symmetry index is a numerical value used to determine the characteristics of the top surface of the cake. While cakes with high symmetry index values have a domed structure, cakes with low symmetry index values have a flatter surface. When the results were examined, it was observed that as the protein isolate ratio increased, the symmetry index values decreased (*p* < .05) (Table 5). There was no statistically significant difference between the symmetry index of the cupcake with 5% protein isolate addition and the symmetry index of the control group sample (*p* > .05). The uniformity index is a measure of the lateral symmetry of the baked cake, and the ideal uniformity index value is zero. When the results were examined, the highest uniformity index value was determined in the control group (*p* < .05) (Table 5). On the other hand, the uniformity index closest to zero was determined in the cupcake sample with a 20% protein isolate addition. Additionally, it was observed that as the protein isolate ratio increased, the uniformity index values approached zero (Table 5).

#### 3.3.3 Moisture content and water activity

The moisture content and water activity values of the samples are shown in Table 6. The increase in protein content caused an increase in moisture content in cupcakes produced with different ratios of protein isolate (Table 6). This observation is believed to be related to the high water‐holding capacity of fenugreek seed protein isolate (Table 3). It was determined that the substitution of 20% protein isolate resulted in a 1.07% increase in the moisture content of the cake. The water activity values of cupcakes varied between 0.862 and 0.880. Although statistically significant differences were observed, the water activity values were generally close to each other (Table 6).

**TABLE 6:** Moisture content, water activity, baking yield, bulk density, and hardness of the cupcakes.

Sample	Moisture (%)	*a*_*w*_	Baking yield (%)	Bulk density (kg/m^3^)	Hardness (*N*)
Control	23.57 (±0.41)^c^	0.880 (±0.005)^a^	82.91 (±0.49)^d^	420.47 (±7.21)^d^	3.44 (±0.04)^a^
PI 5%	23.59 (±0.30)^c^	0.879 (±0.006)^a^	83.55 (±1.06)^c^	440.38 (±2.55)^c^	3.10 (±0.05)^b^
PI 10%	24.20 (±0.15)^b^	0.866 (±0.002)^b^	83.75 (±0.65)^bc^	462.08 (±4.51)^b^	2.60 (±0.04)^c^
IP 20%	24.64 (±0.30)^a^	0.862 (±0.004)^b^	84.33 (±0.43)^ab^	504.63 (±6.25)^a^	2.36 (±0.05)^d^

*Note*: ^a–d^Means with unusual superscripts within a column are significantly different (*p* < .05).

Abbreviation: PI, Protein isolate.

#### 3.3.4 Baking yield, bulk density, and hardness

The baking yield, bulk density, and hardness values obtained for cupcakes are also presented in Table 6. It was determined that the baking yield values for protein isolate‐added cupcakes and the control group varied between 82.91% and 84.33%. Although similar results were obtained in terms of baking yield, the highest baking yield was obtained in the sample with 20% protein isolate replacement (*p* < .05) (Table 6). When the bulk density values of cupcakes were examined, it was observed that as the protein content increased, the bulk densities also increased (*p* < .05) (Table 6). While the highest bulk density value was found in the cupcake with 20% protein isolate, the lowest bulk density value was observed in the control sample (*p* < .05) (Table 6). Hardness (N) is defined as the maximum force applied to the compressed sample (Majzoobi et al., 2014). According to the results obtained in the study, as the protein content increased, the hardness values of the samples decreased (*p* < .05) (Table 6).

## 4 CONCLUSION

This study revealed the potential of ultrasonic‐assisted extraction, a green extraction method, for maximizing protein extraction from fenugreek seeds. The extracted proteins have remarkable functional properties and significant nutritive value, making them ideal for food applications, including as emulsifiers, foaming agents, and stabilizers. Furthermore, the molecular weights, amino acid composition, and structural properties of the protein isolates were elucidated, providing a comprehensive understanding of their physicochemical properties and highlighting their nutritional value. The findings contribute to the exploration of sustainable approaches for plant‐protein extraction and pave the way for the development of innovative food products with enhanced nutritional profiles. Incorporating fenugreek seed protein isolate into cupcake formulations improved key physical attributes of cupcakes. Cupcakes enriched with protein isolates showed an increased browning index (BI) compared to the control sample, while moisture content and water activity remained consistent across all samples. Evaluation of volume, symmetry, and uniformity indexes indicated smoother external structures in cupcakes containing protein isolates, suggesting potential improvements in product aesthetics. Furthermore, increasing protein content correlated with decreased hardness values, indicating potential textural enhancements. Consequently, this study also underscores the multifaceted impact of fenugreek seed protein isolate on cupcake quality and characteristics, highlighting its potential as a functional ingredient in bakery products.

## AUTHOR CONTRIBUTIONS

**Izzet Turker:** Data curation (equal); formal analysis (equal); investigation (equal); writing – original draft (equal). **Gamze Nur Olgun:** Data curation (equal); formal analysis (equal). **Hilal Isleroglu:** Conceptualization (equal); methodology (equal); supervision (equal); writing – original draft (equal); writing – review and editing (equal).

## CONFLICT OF INTEREST STATEMENT

None.

## Data Availability

The data that support the findings of this study are available from the corresponding author upon reasonable request.
